# Delusions of Possession and Religious Coping in Schizophrenia: A Qualitative Study of Four Cases

**DOI:** 10.3389/fpsyg.2021.628925

**Published:** 2021-03-19

**Authors:** Igor J. Pietkiewicz, Urszula Kłosińska, Radosław Tomalski

**Affiliations:** Research Centre for Trauma and Dissociation, SWPS University of Social Sciences and Humanities, Katowice, Poland

**Keywords:** religious delusions, possession, cenesthetic hallucinations, hearing voices, religious coping, exorcisim

## Abstract

The notion of evil spirits influencing human behavior or mental processes is used in many cultures to justify various symptoms or experiences. It is also expressed in psychotic delusions of possession, but there is limited research in this area. This study explores how patients with schizophrenia came to the conclusion that they were possessed, and how this affected help-seeking. Interviews with two men and two women about their experiences and meaning-making were subjected to interpretative phenomenological analysis. Three main themes were identified: (1) Links between traumatic experiences and psychotic symptoms, (2) The emergence of religious themes in delusional contents, and (3) Reluctance to use medical treatment and instead to seek exorcism. In each case, attributing problems to possession was supported by the local environment and media, led to seeking spiritual help, and delayed diagnostic assessment and treatment. However, using religious coping contributed to the sense of predictability and social support. Clinicians are encouraged to explore the experiences and conflicts expressed by the symptoms which people ascribe to possession and to negotiate alternative explanatory models with their patients.

## Introduction

Patients' spiritual involvement and the religious context of their lives is likely to affect clinical presentation and the content of psychotic symptoms (Bhavsar and Bhugra, 2008; Gearing et al., 2011; Cook, 2015). This cultural pathoplasticity can be seen in different studies; for instance, Protestants report religious delusions more frequently than Catholics (Getz et al., 2001), and being a Catholic is more conducive to delusions of sin and guilt compared to Muslims (Stompe et al., 2006). Cook (2015) estimates that 20–60% of patients with psychosis report delusions with religious content. Gearing et al. (2011) identify three types of religious delusions or hallucinations: (1) religious themes, (2) religious figures, and (3) supernatural. The first category includes direct references to organized religious themes, including prayer, sin, or possession. The second relates to the presence of religious figures such as God, Jesus, devil, or prophet. The third includes more general mystic references: black magic, spirits, demons, being bewitched, ghosts, sorcery, voodoo.

There is limited research on delusions of possession, which can be viewed as a sub-category of religious delusions. In this study, delusion of possession will be understood as distorted perception of having one's mental processes or actions controlled by supernatural powers (e.g., devil, spirits, Satan, Lucifer, Jesus, and Virgin Mary). This would distinguish delusions of possession from other forms, where individuals identify themselves as religious heroes or maintain that they possess supernatural powers. There is limited research on delusions of possession but some authors estimate their presence in 20–40% of psychotic patients, more often in women than men (Iida, 1989). Kopeyko et al. (2018) stress that they can be accompanied by other symptoms: delusions of influence or hypochondriacal, cenesthetic or olfactory hallucinations, depressive mood, and suicidal tendencies. Patients with these symptoms also report more childhood sexual trauma, have higher dissociation scores, more cannabis abuse, experiences of thought control, and hear more “voices” inside their heads (Goff et al., 1991). Different authors stress that the content of delusions may be a direct reconstruction of traumatic experiences or indirectly refer to them, as decontextualised memories (Corstens and Longden, 2013; Hardy, 2017; Moskowitz et al., 2019; Peach et al., 2019).

Interestingly, all symptoms mentioned earlier are also frequently present in people with dissociative disorders (Van der Hart et al., 1996, 2006; Howell, 2011; Pietkiewicz and Lecoq-Bamboche, 2017). About 50% of dissociative cases also meet criteria for schizophrenia, which is one reason why 27–41% of them were initially diagnosed with psychosis (Renard et al., 2017). Ross (2018) also presents a concept of dissociative psychosis arguing that some patients with psychosis may have comorbid dissociative symptoms. From the clinical perspective, however, it seems reasonable to differentiate between psychotic and dissociative structures because different types of treatment will be recommended for them (Moskowitz et al., 2019). Thus, clinicians need to distinguish delusions of possession *per se* from other symptoms common in dissociative disorders, namely passive influence or intrusions of dissociative parts (Van der Hart et al., 2006). On the phenomenological level, some cenesthetic or olfactory hallucinations can also resemble trauma-related somatoform dissociation. Thus, understanding the quality of reported experiences in the context of accompanying symptoms, and how they are being reported by patients, is helpful in differential diagnosis.

Kopeyko et al. (2018) also stress that delusions of possession is a predictor for unfavorable prognosis and treatment. This may result from the fact that people who are convinced they are possessed are more likely to seek help from priests than healthcare providers. According to Goff et al. (1991), delusions of possession can develop when it is suggested to people in psychosis that their symptoms are supernatural. In an Indian study, 40% of schizophrenic patients were encouraged by their families to accept such interpretations and participate in *magico-religious treatment* (faith healing) instead of consulting a psychiatrist (Kulhara et al., 2000). This may be because being possessed in that cultural context is less stigmatizing than having schizophrenia (Ventriglio et al., 2018). Unfortunately, it also discourages or delays psychiatric treatment and psychotherapy. However, similar phenomena have also been observed in developed countries like Poland (Pietkiewicz et al., 2021), Spain (Tajima-Pozo et al., 2011), or the UK (Leavey, 2010). Pietkiewicz et al. (2021) stress that religious leaders can influence help-seeking pathways and either refer people for clinical consultations or strengthen the belief in being possessed and encourage exorcisms. Tajima-Pozo et al. (2011) illustrate that with a case study of a 28-year old patient treated with medication and psychotherapy for paranoid schizophrenia with persistent delusions of possession. Because her treatment did not reduce cenesthetic hallucinations, priests led her to believe that her symptoms were due to the presence of a demon.

This study analyses the experiences of possession in patients diagnosed with schizophrenia. It explores how they came to the conclusion that they were possessed, how they reported their symptoms, and what coping strategies they used.

## Materials and Methods

This study was carried out in Poland between 2018 and 2020. Qualitative data were gathered using in-depth clinical interviews and psychiatric mental health assessments, and were subjected to Interpretative Phenomenological Analysis (IPA). IPA is a popular methodological framework in psychology, based on the principles of phenomenology, hermeneutics, and idiography (Smith and Osborn, 2008). It explores participants' experiences and interpretations, followed by researchers trying to make sense and comment on these interpretations (“double hermeneutics”). Samples in IPA studies in particular use small, homogenous, purposefully selected, and qualitative material which is carefully analyzed case-by-case (Smith and Osborn, 2008; Pietkiewicz and Smith, 2014).

IPA was chosen for this research to analyse how patients diagnosed with schizophrenia decide they are possessed. It qualitatively explores their symptoms and their links with stressful life events. This study illustrates how exorcism seekers diagnosed with schizophrenia are distinct from other individuals who engage in religious rituals because they think they are possessed, but meet criteria of personality disorders and dissociative disorders.

### Procedure

This study is part of a larger project examining phenomena and symptoms reported by people seeking religious rituals to liberate themselves from demonic influence. This project was held at the Research Center for Trauma and Dissociation, financed by the National Science Center, and approved by the Ethical Review Board at the SWPS University of Social Sciences and Humanities in Poland. Potential candidates enrolled themselves via an application integrated with a dedicated website, or were registered by healthcare providers and pastoral counselors. They filled in demographic information and completed online tests, including: Somatoform Dissociation Questionnaire (SDQ-20, Pietkiewicz et al., 2019a), Dissociative Experiences Scale - Revised (DESR, Pietkiewicz et al., 2019b), and Traumatic Experiences Checklist (Nijenhuis et al., 2002). They then participated in a semi-structured interview exploring their biography, family situation, religious socialization and spiritual involvement, and motives for enrolling in the study, followed by an in-depth clinical assessment investigating their symptoms. This interview was followed by an additional mental state assessment performed by the third author, who is a psychiatrist. He collected medical data, and with people who reported psychotic symptoms he double-checked them using PANNS (Kay et al., 1989), confirmed and communicated the diagnosis and discussed available coping strategies. All interviews were video-recorded and assessed by three healthcare professionals, who discussed each case and consensually reached a diagnosis based on ICD-10. Four participants out of 17 who enrolled in the project were selected for this IPA study. Selection was based on the following inclusion criteria: (a) labeled in local communities as possession victims, (b) made contact and used the support of exorcists, (c) met criteria for schizophrenia. Interviews with every participant in this study ranged from 1 to 4 h.

### Participants

Participants in this study were two men and two women, all Roman Catholics, aged between 21 and 30 years, diagnosed with schizophrenia, and labeled by members of local religious communities or families as “possessed” due to their odd behavior, auditory, and cenesthetic hallucinations. Two had secondary education, and two of them primary. None of them worked, three lived with their families and were sustained by them and one was a resident of a social welfare home. All participated in religious activities and made contact with exorcists because they were convinced they were possessed. Two were subjected to multiple individual exorcisms, and two used deliverance prayers. Two read about the study on the website and enrolled themselves, one was referred by a psychiatrist, and one by an exorcist. All had previously been hospitalized due to psychotic symptoms and responded well to treatment. All, except Kathy, had the onset of psychotic symptoms after the age of 20. All were socially withdrawn, reported different schneiderian symptoms and problems with affect regulation. In their childhood, all participants suffered from emotional neglect, physical or sexual abuse. However, none of them had symptoms of PTSD, amnesia, or symptoms indicating the existence of distinct identities. They had elevated scores in SDQ-20 (above 28 points) indicating some somatoform dissociation and in DESR (above 72 points) indicating some psychoform symptoms, but none of them met criteria for a dissociative disorder. For information about the participants see Table 1. Their names have been changed to protect their confidentiality.

**Table 1:** Study participants.

Kathy	Age 21, single, primary education, unemployed and has never worked. She tries to develop artistic talents (painting, poetry, music). Lives with her mother and her mother's partner who is emotionally abusive toward her. Parents divorced and she has no contact with her father or elder brother, both of whom had problems with alcohol and were violent. Reports domestic violence for 10 years since she was eight. She has no friends and has never had an intimate relationship. She has been hospitalized multiple times for schizophrenia since she was 14. She did not comply with her treatment regimen but never abused any substances, and was never suicidal. Reports cenesthetic hallucinations, delusions of influence, grandiose and religious delusions (telepathic communication with Jesus Christ and Lucifer, and delusions of possession) and thoughts broadcasting. She consulted priests and exorcists hoping they would support her mission to redeem demons. Referred for a diagnostic assessment by the exorcist. She enrolled in the study to receive an opinion from a professional and instruct others how to save demons. Test scores: SDQ-20: 28, DESR: 81.
Alice	Age 30, single, secondary education, unemployed, used to work in a supermarket. She has been in a relationship for 6 years. She lives with her parents and her partner, although her mother is absent for weeks due to work; both men abuse alcohol. Experienced bullying at school. In adulthood, she frequently drank alcohol and sometimes used drugs (amphetamine, methamphetamine, marihuana, legal highs). She also experienced physical and emotional abuse while working as a prostitute for a few years. She has no friends and avoids social contacts. Since age 28, hospitalized multiple times for schizophrenia. Reports auditory and cenesthetic hallucinations, and delusions of possession, influence and grandeur. She used individual exorcisms regularly for 18 months. Referred to the study by a psychiatrist. Test scores: SDQ-20: 32, DESR: 50.
Charles	Age 25, single, secondary education. Unemployed, used to work as a manual worker in Poland and abroad but stopped due to his symptoms. Lives with his mother, her partner and younger brother. He has no friends, no partner. He used to have casual, sexual relationships with women. Parents divorced because his father abused alcohol, drugs, was verbally and physically violent. He also reports unwanted sexual experiences with his grandmother and cousin when he was 10. Has abused marihuana since he was 15 and convicted for driving under the influence of drugs. Had two suicidal attempts. At age 22, diagnosed with schizophrenia and hospitalized three times. Reported delusions of possession, reference, persecutory, and grandeur, thoughts broadcasting, auditory and cenesthetic hallucinations. Consulted an exorcist a few times and participated in deliverance prayers. Enrolled in the study hoping to rule out a mental disorder. Test scores: SDQ-20: 34, DESR: 21.
Greg	Age 30, single, primary education. Unemployed, used to work at a construction site or distributed leaflets. His parents divorced when he was young. His parents abused alcohol, mother was also physically violent. He abused marihuana and methamphetamine between ages 15–23. Hospitalized multiple times due to aggression toward strangers and drugs. Reported persecutory delusions and delusions of reference and possession, thoughts broadcasting. Feels compelled to make strange gestures or movements. Denied having hallucinations. Diagnosed and treated for schizophrenia with little improvement. Taken by mother to exorcists who often restrained him with leather straps. Following his mother's death, he had problems with daily functioning and after a suicide attempt (jumping off a bridge), he was placed in a social welfare home 3 years before the interview. He has no friends, has been involved in a Christian charismatic movement and remains in touch with an exorcist. He hoped the study would confirm that he was possessed. Test scores: SDQ-20: 49, DESR: 153.

### Data Analysis

All video recordings were transcribed verbatim and analyzed, together with researchers' notes, using qualitative data-analysis software – NVivo10. Consecutive steps recommended for IPA were employed in the study (Pietkiewicz and Smith, 2014). For each interview, researchers watched the recording and carefully read the transcript several times. They individually made notes about body language, facial expressions, the content and language use, and wrote down their interpretative comments using the annotation feature in NVivo10. Next, they categorized their notes into emergent themes by allocating descriptive labels (nodes). The team then compared and discussed their coding and interpretations. Connections between themes in each interview and between cases were analyzed, and grouped according to conceptual similarities into main themes and sub-themes.

### Credibility Checks

During each interview, participants were encouraged to give specific examples illustrating reported symptoms or experiences. Clarification questions were asked to negotiate the meaning participants wanted to convey. At the end of the interview, they were also asked questions to check that their responses were thorough. The researchers discussed each case thoroughly and also compared their interpretative notes to review their understanding of the content and its meaning (the second hermeneutics).

## Results

Participants described their symptoms and meaning-making. Salient themes emerged in interviews and were organized into three main themes and two sub-themes, as listed in Table 2. Each theme is discussed and illustrated with verbatim excerpts from the interviews, in accordance with IPA principles.

**Table 2:** Main themes and sub-themes identified during the analysis.

Theme 1: Links between traumatic experiences and psychotic symptoms
Insecure and unpredictable environment
Deprived of close relationships and attention
Theme 2: The emergence of religious themes in delusional contents
Theme 3: Reluctance for medical treatment, and seeking exorcism

### Theme 1: Links Between Traumatic Experiences and Psychotic Symptoms

Participants reported traumatic experiences in close relationships, from their childhoods until the present. The content of their psychotic symptoms seemed to be connected to these experiences and conflicts related to expressing anger, sexuality, and dependency needs.

#### Insecure and Unpredictable Environment

All four suffered physical violence and emotional abuse from one or both parents. They had not necessarily linked this with their symptoms although certain patterns became apparent during the interviews. Delusions and hallucinations seemed to help the women restore a sense of strength and control, especially when faced with the aggressive behavior of family members. Kathy said her parents abused alcohol and quarreled, and her elder brother insulted and hit her. When she was 14, she started talking with a warrior in her head, who instructed her how to become powerful like him.

They were violent. My mother's partner used to call me a “cunt” or “lunatic.” This Mayan warrior helped me. I discovered during meditation that we had met in a previous life. He always made comments about my behaviour. I wanted to gain weight and prepare myself to be like a Mayan warrior myself (Kathy).

She also found solace wandering around forests and meadows, thinking she could communicate with animals. To reduce emotional tension, she ran away from home naked with a duvet wrapped around her. A few times she released birds from cages or broke into someone's house to take a bath, which she justified in a delusional way.

I could always talk to animals. I went to a nearby forest and talked telepathically with a roe deer. I asked her to find me a man with a dog, who I saw in a vision from my previous incarnation [.] I had to release birds which farmers kept in cages because they asked me to set them free. [.] One woman told me I should purify myself, so I tried to take a cleansing bath. But the police came and took me to hospital. I wanted to go to the hospital to meet a man from my vision. Its principle was resonance, a divergence between content and the universe (Kathy).

Alice did not feel supported by her family when she was bullied at school. Her mother was absent because she often went abroad to work as a caregiver, leaving Alice with her partner and father, both of whom were alcoholics, quarreled and were often abusive toward her. She earned money as a prostitute and was also badly treated by her clients. She took drugs offered by a client, and started hearing voices of different people she met in everyday life. These voices comforted her and confirmed her clairvoyant abilities.

These voices would tell me that someone would soon die, and this would really happen. They said I would get worse, hear more voices and feel them inside. They foretold I would be taken to a psychiatric asylum and indeed I ended up in a mental health unit (Alice).

The male participants experienced their symptoms differently. They felt persecuted and unable to control internal hostile impulses, which they thought were alien. This strengthened their anxiety. Greg felt he was being watched and ridiculed by everyone and even physical objects or animals were hostile toward him. Subsequently, he would avoid people or become vulgar and violent.

I need to fucking hide because they eavesdrop on me all the time. They don't let me fucking live! What the fuck, fuck, fuck. Fuck them all. This pisses me off so much. You pricks and whores. I need to beat someone up (Greg).

His constant vigilance can be understood in relation to childhood traumas, because he was brought up by an alcoholic mother who was controlling and violent toward him.

My mother was very impulsive, physically aggressive. She behaved crazy sometimes. Once she took a knife and tried to stab me in my belly. There was something really wrong with her. Sometimes she behaved like me (Greg).

Charles experimented with different drugs in early adulthood and started having auditory hallucinations accompanied by physical sensations. The voices insulted him and dared him to be more masculine, meaning: bold, arrogant, making sexual approaches to women.

The voices call me “faggot,” ‘loser” or “cunt.” They want me to start thinking with my penis. Wherever I go or whatever woman I see, they want me to flirt with her and have sex […] They tell me I should be brutal, call these girls “sluts” or “my personal bitches” (Charles).

To his surprise, he discovered that the content of his voices exactly reflected attitudes and even phrases used by his father, uncle and cousin – men who represented important role models and also became the objects of his loathing, who frequently verbally and physically humiliated him. His auditory hallucinations were usually accompanied by somatic sensations: he had a feeling that someone touched him or blew air on his skin, especially intimate areas. Before one of his hospitalisations, he felt he was being raped by the voices, although he admitted this experience was thoroughly incomprehensible.

There is a female voice, sort of touching me and I get aroused. I feel this touch as if it was an illusion of oral sex. This is not real but I can clearly feel it. Male voices say they are perverted and will do with me whatever they want. One night, I could feel something in my anus, like a ghost penis. They raped me and I felt their cum on buttocks when they finished (Charles).

Asked about his associations, he talked about his drunk grandmother who fondled his penis when he was 8 years old. At the age of 10, his 2-years-older cousin encouraged him to have sex. Memories of performing fellatio or being penetrated evoked conflicting emotions in Charles and made him question his masculinity. He said he always felt compelled to prove his heterosexuality in adult life and remained extremely resentful toward his cousin, referring to him as a pedophile who raped him.

#### Deprived of Close Relationships and Attention

In adult life, participants tended to isolate themselves and had limited social interactions, despite longing for meaningful relationships. Their social contacts usually involved immediate family, or caregivers working in a social welfare home (in Greg's case). The women emphasized feeling lonely and unimportant. Kathy hoped that her artistic family would appreciate her own talents, so she danced in front of them eagerly or showed them her poems, paintings, or YouTube videos. However, she was convinced that everyone perceived her as weird and maladjusted. She may have developed her belief in supernatural telepathic powers or her perceived ability to communicate with aliens in order to cope with loneliness.

These are extra-terrestrials from a different dimension, another frequency band. We have had contact for 3 years now. They can be quite intrusive. We communicate telepathically. I can hear and feel them. Sometimes we even have intercourse (Kathy).

Shortly before the onset of auditory hallucinations, Alice lost two important women in her life – her aunt, who overdosed on alcohol, and a friend, who hanged herself after taking drugs. Faced with loss, Alice started hearing their voices, and they became her companions, comforting her and helping in making decisions.

This friend became even closer to me after her death because I could hear her voice in my head [.] These two voices told me how to live and what I should not do. For example, they instructed me to take loans and move away from home. So I ended up in debt, but my father paid everything (Alice).

The male participants said they preferred to withdraw from social life, convinced that their symptoms were weird, embarrassing or dangerous. After his mother's death and a suicide attempt, Greg was sent by the court to a social welfare shelter for the homeless and mentally ill. He had no friends and his biological father refused to care for him. Greg grew more anxious, hostile, had delusions of reference, and could not resist making strange hand movements imitating rap singers or bending down while walking.

I was half bent over, and walked in this way. I couldn't control it and people commented about it. They said I must be ill or fucked up. They paid attention to me because I behaved weird (Greg).

Charles thought he should refrain from social interaction until his condition improved. Although he felt attracted to a girl, he was afraid of harming her feelings or being rejected for his symptoms or past experiences.

I tell myself that I am ill and do not want to mix with other people. If I get back in shape mentally, I will renew my relationships. There is a woman I fancy, an old friend of mine, but I do not see her. I don't want to hurt her and I also find it difficult to talk about what happened in my past (Charles).

He experienced conflicts related to his own identity: he disapproved of the behavior of the men who were his only role models, and also questioned his masculinity and heterosexuality.

### Theme 2: The Emergence of Religious Themes in Delusional Contents

Initially, religious motives were not present in their delusional experiences, but emerged under the influence of the environment. They were inspired by cultural beliefs and evolved as participants' interpretations of unusual experiences. When her father moved out following a divorce, and domestic violence stopped, Kathy started sharing his interests in the occult and read his books. She thought spirits influenced her behavior and convinced herself she had gained special powers in order to save demons. Subsequently, she developed and performed purification rituals.

I often scream at home with the voices of these demons, because I have to purify them. I have to connect to them energetically and use a special prayer for cleansing them of their sins. I developed this method based on my own observations, or films like “The Exorcism of Emily Rose.” [.] These spirits went to hell, which I think is more of a mental realm. They simply did something, killed a family member or were cruel, and later on they felt guilty for their actions, and this condensed in a single unit of time (Kathy).

Alice initially heard positive voices of her deceased friends or people she had recently met in real life. When she was told that these auditory hallucinations were demons, the voices proliferated and some became distressing, so she wanted to get rid of them.

I met a meditation instructor who said they were demons who wanted something from me. I got really scared. Not only did I hear these ghosts but I also started hearing demons… and I could see them as well… Before I was admitted to hospital I heard countless voices and it was really scary, because they were demons. Later I thought I was hearing angels when someone gave me a prayer to Archangel Michael. When I felt that something was touching me, I would pray to him and this sensation stopped (Alice).

Greg was hospitalized during psychotic episodes numerous times. He said he was “a dangerous psychopath with murderous instincts.” While doctors diagnosed him with schizophrenia, his mother justified his paranoia and anger attacks with demonic possession. She educated him about exorcisms and made him participate in such rituals during his leave of absence days. Finally, Greg accepted her interpretations and endorsed the notion of the demonic evil forcing him to make strange gestures, being hostile and violent. This filled him with awe but also gave a sense of understanding of his symptoms.

My mother said I was controlled by evil. She read to me about people who were possessed. She had a book written by Father Amorth, a famous exorcist. It is scary to think that I am possessed by demons. I actually feel terrified. On the other hand, there is no other explanation for all these things I have experienced. [.] When I had skip days, she took me from hospital and we visited exorcists. I must have performed about 20 rituals (Greg).

Charles's explanatory models of his unusual experiences changed over time. Initially, he was convinced he had a chip in his brain after reading on the Internet about alien civilizations controlling humans. He even saved money for a computed tomography (CT) scan to confirm his theory.

These voices convinced me that I had a chip installed in my brain. I had a sensation in my head, like an electric current, an electronic impulse. I also read about it on the web. I had to find out if I was mentally ill or had an implant, but the tomography did not prove anything (Charles).

Because his voices and tactile symptoms persisted despite pharmacotherapy, he also refused to accept the medical diagnosis of schizophrenia, deciding instead that his problems could only be explained in terms of demonic possession. He believed that spirits could produce symptoms resembling mental illnesses and could also mess with his mind.

I also read that some evil spirits can imitate mental illness. I don't know if that is the case because I never had problems with my faith. But I read that these spirits feed on human weakness. (.) These are spirits. It is hard to explain. Three of them are responsible for my symptoms. There is a female voice and two male voices. In general, they say they are Virgin Mary, Jesus Christ and God. I don't believe them because God would not interfere with someone's mind. How can I believe that God or Jesus would use [rape] me in such a tragic way. They constantly comment on my actions and judge me. They have no insight into my heart, how I feel and what I am really like (Charles).

### Theme 3: Reluctance for Medical Treatment, and Seeking Exorcism

All participants were referred to priests, because families or communities labeled them as possessed. Kathy said that her mother had tried to help her get rid of spirits by taking her to a tarot reader, pranic healer, and a hypnotist. She was later consulted by an exorcist called by nuns. Kathy admitted she did not expect him to perform any rituals. Instead she wanted to teach the exorcist that his view about demonic beings was wrong, and to demonstrate her auto-exorcism skills.

After visiting all these practitioners, I went to visit nuns. I thought it would be so extraordinary if demons could pray together with them but they panicked and called an exorcist. They were scared because I articulated these demons. I had to set them free to purify them. The content was actually not that bad but the sound terrified them. It was demonic. [.] So I went to that exorcist to teach him about spirits. He meets so many of them but knows so little. I wanted to inform him about the real nature of evil, that there is nothing to be afraid of. If he had accepted that, I could have helped him in his services but he just sent me here. He said he had to make sure I did not have any mental issues (Kathy).

Alice, on the other hand, attended regular monthly exorcism rituals for 18 months. She said that she managed to appease the distressed demonic voices but she “opened the gate for Lucifer” to possess her.

I was attending exorcisms and this priest told me that Satan was messing with my head. He used his book of demons and prayed over me. He commanded them to leave my body, and I felt something leaving. At the same time, something else entered. This was probably Lucifer… After these rituals I stopped hearing the demons and only Lucifer remained. I think he helped me by making the other spirits disappear. He is an angel and has great power. He is so strong. I also heard voices of different angels, for example Archangel Michael and the Angel of Death (Alice).

Her relationship with the new voice was dynamic and changed over time. While initially Lucifer's voice caused distress, she later found his company comforting and reassuring. It also helped her control her aggressive impulses.

Lucifer is bad – that is what I thought at first, so I fought against him and was really scared, like I was scared of the demons. But later things changed. I realized that if I cannot get rid of him, I need to collaborate with him and things became different. At first I thought he wanted to take the [aunt's and friend's] ghosts with him, and I did not want to let them go. I didn't want to let him harm them. I was not afraid anymore. [.] Lucifer says I will go to the city of angels because I am the Angel of Balance. He says I should discriminate between good and evil and refrain from any bad actions or he will punish me. He says that taking drugs or alcohol is bad, and that I should be kind to others (Alice).

Because her exorcist did not approve of the voice, which represented to him demonic influence, Alice decided to stop the exorcisms and terminate her relationship with the priest. She also tried to develop her own understanding of the symptoms independently from the explanatory models of priests and doctors.

I stopped attending these rituals because I am happy that I can hear Lucifer, and the other voices are gone. I do not need that priest any more really. According to him, Lucifer is bad. He has his own theory, the doctor has his own, and I have mine < laughs>. I think I have a mental illness but I also have supernatural experiences (Alice).

After Greg's mother died, he continued meeting exorcists, who became one of his very few sources of support. He also got involved in a charismatic Christian movement which enhanced his belief in demonic possession. He denied having a mental illness and was reluctant to take medicines prescribed by his doctor or participate in psychotherapy sessions offered at the social welfare home. However, he also admitted that not all exorcists agreed that he was really possessed.

Initially, they were certain about my possession because I behaved weird. Others said they had doubts about that because they used the holy water and it did not work. They said that if the exorcized water and prayers have no effect, it means I am not possessed (Greg).

Participating in exorcisms became impossible when he was incapacitated in a social welfare shelter. After that he could only attend prayers with his exorcist on the phone. Greg liked talking to priests who were friendly and interested in his story, but did not enjoy meeting exorcists who were harsh or restrained him.

Some meetings with exorcists were based on a kind of investigation about what is wrong with me. Some of them were really nice, kind-hearted. At least, this is how I saw them. Others were kind of harsh, radical. They used leather belts or called other men to restrain me (Greg).

To confirm his theory about possession, Charles also consulted an exorcist. The priest did not refute his ideas and encouraged regular meetings to investigate that possibility. On the other hand, he also suggested using deliverance rituals to cleanse Charles of demons and evil spirits.

I visited the exorcist and we talked. I wanted to know whether these experiences resulted from the influence of an evil spirit or not. I just wanted to rule out spiritual factors. If he rejects that, then I can be 100% sure this is some kind of internal flaw, mental problem. He neither confirmed nor disconfirmed. Instead, he suggested doing these prayers (Charles).

Being touched by the priest seemed to evoke in Charles aggressive impulses which could have been related to his earlier memories and conflicting feelings.

I told him about the voices in my head. He then put his hand on my head and started to pray. I remember that very well because the voices got very upset and continuously repeated: “You prick!” Both male and female voices said: “This priest is a pedophile.”

Charles was too ashamed to tell the priest what he was feeling or hearing, fearing that, if he did, it may have been interpreted as aversion toward the sacred, an indicator for the exorcist that he was probably dealing with a possessed individual.

## Discussion

This study illustrates how people with psychosis can develop a strong conviction that they are possessed. There are both similarities and differences between this group and other individuals who are labeled in local communities as “possessed,” but present features of personality disorders (Pietkiewicz et al., 2021) or dissociative disorders (Van der Hart et al., 1996; Pietkiewicz and Lecoq-Bamboche, 2017). The common feature is the belief in being possessed, which is co-created by the social environment. This shared belief is usually linked to possession-form presentations, described by Van Duijl et al. (2013) in terms of alterations in behavior and consciousness, marked by: talking in a different voice, sensation of paralysis, shaking, glossolalia or making animal sounds, fugues, or “night dances.” However, the notion of possession can be stretched to explain problems with affect regulation, unaccepted impulses, difficulties in spiritual practice, or even unpleasant events in the family (Pietkiewicz et al., in print). Additional indicators of demonic influence may also include: exposure to inappropriate music or films, using substances, masturbation, homosexuality or extra-marital sex.

From the psychiatric perspective, the belief in being possessed can be qualified as an overvalued idea – an unreasonable and sustained belief which is less intense than a delusion, i.e., the person is able to acknowledge the possibility that the belief may or may not be true (Veale, 2002). Delusional convictions, one the other hand, are traditionally considered as more rigid and unquestionable in nature. People with delusions are also less functional in daily life, compared to those who simply have overvalued ideas (Veale, 2002). This study shows, however, that there may be a blurred line between an overvalued idea and delusion of possession. For example, one participant (Charles) did not show extreme rigidity about his belief in possession but sought possible explanations for his incomprehensible experiences. Their social context, which normally helps in reality testing and provides reference points for delusions, only strengthened participants' beliefs about their possession. For this reason, it can be hypothesized whether the belief in possession described here can be regarded as a shared delusion or a shared overvalued idea.

When the social context fails to provide good reference points, it is necessary to rely on other psychopathological symptoms to determine the psychotic nature of people's convictions: the existence of other delusions (messianic, grandiose, persecutory) or auditory and tactile hallucinations. Participants in this study had various delusional interpretations of the external world and their internal experiences, e.g.,: a conviction that she is the Angel of Balance, having a mission to save spirits, communicating telepathically with animals and extra-terrestrial beings, being controlled through a chip in the brain, or persecuted by people, animals and objects. Generally, such symptoms are not to be expected in other disorders.

While auditory hallucinations may be present in all three diagnostic categories mentioned earlier (Longden et al., 2019; Moskowitz et al., 2019), the qualitative features of “voices” may differ in these disorders (Dorahy et al., 2009). In this study, voices were very simple, non-responsive, sometimes personified the abusers or were exact copies of what they previously said, but the link to the context was not obvious. This supports other observations that psychotic symptoms may directly or indirectly (thematically) relate to traumatic experiences (Hardy et al., 2005; Corstens and Longden, 2013; McCarthy-Jones and Longden, 2015). While these features do not necessarily distinguish patients with psychosis, characteristic to this group were the delusional interpretations of auditory hallucinations.

Kopeyko et al. (2018) also observed that delusions of possession are sometimes accompanied by cenesthetic hallucinations and mood problems. Similar symptoms were reported in this study, although the quality of somatic experiences was different among participants. Both women mentioned a strange, partially pleasant and arousing “tingling sensation in the tummy” which they ascribed to spirits communicating with them. On the other hand, symptoms reported by Charles (feeling someone blow air on his skin, touch his genitals, perform fellatio, penetrate his anus or ejaculate on him) were directly related to situations experienced by him as traumatic and causing strong intrapsychic conflicts related to identity (masculinity, heterosexuality). Some clinicians could classify these symptoms as somatoform dissociation and not cenesthetic hallucinations. In other words, Charles could be re-experiencing in the present the somatic aspects of his traumatic memories, but his interpretations were again delusional, i.e., “Jesus or God raping me.”

This study also shows that patients' explanatory models about their symptoms are dynamic and evolving, especially under the influence of media (Facebook, YouTube, books), family, and the religious community (priests and other practitioners). Delusions of possession are embedded in folk beliefs about the influence of evil spirits. When other people, e.g., priests or family members, shared similar beliefs, this only reinforced delusional thinking, discouraged the use of diagnostic consultations and negatively affected compliance. Perhaps, as Ventriglio et al. (2018) say, it is more acceptable to be “possessed” than have a psychiatric disorder because it relieves people of the stigma associated with mental illness. From the point of view of the recovery movement, being part of a spiritual community and finding socially acceptable labels for one's problems gives empowerment and social inclusion which may outweigh possible drawbacks (Jacob, 2015). This may be particularly true for people with psychosis rather than other disorders. Alice and Greg, for example, had stable, long-term relationships with priests who not only provided them with emotional support but also structured their daily activities. On the other hand, relying solely on religious coping can in some cases delay diagnosis and professional treatment, which may lead to worse outcomes in people with psychosis (Albert et al., 2017).

In Polish society, values, beliefs and norms are strongly influenced by the Roman Catholic Church. Reporting possession and seeking exorcisms is fairly common but official statistics is missing. In their decree, bishops encourage exorcists to refer such petitioners for clinical assessment before offering them any services. However, there is no evidence on how often this recommendation is respected because exorcisms have become a gray area of practice (Pietkiewicz et al., 2021). Giordan and Possamai (2017) observe similar phenomena in Italy and emphasize difficulties in obtaining reliable evidence. There is need for further studies exploring the prevalence of delusions of possession and how people use exorcisms in different cultural contexts. Such studies are especially necessary in developed countries, among people representing different denominations or declared non-believers.

## Limitations

The basic limitation is the number of participants but IPA studies are, by nature, limited to small samples in order to elaborate people's individual experiences and meaning-making. The second difficulty was to reach out to people who are labeled as “possessed” and participate in exorcisms. Some respondents who were recommended by priests or doctors to participate in this study reported ambivalence about using clinical assessment and receiving a diagnosis. Reluctance to refer people to healthcare providers who did not openly declare endorsement and identification with Catholic beliefs could also be found among some exorcists. Furthermore, different results might be obtained from people with delusions of possession who have never been exorcized or live in other cultural contexts. Finally, despite the fact that all participants in this study reported traumatic experiences, quantitative analyses are necessary to investigate links between trauma and delusions of possession.

## Conclusions

Delusions of possession are a separate sub-category of religious delusions in psychosis. They involve a distorted perception of having one's mental processes or actions controlled by demons or spirits associated with local religion. Hearing voices, having incomprehensive bodily sensations, and thought control was attributed to this influence. Other psychotic symptoms were also present but no rapid changes in behavior or identity were reported. Beliefs in possession were induced or strengthened by family, clergy or media, and delayed diagnostic assessment and treatment. Instead, participants chose religious coping strategies, including individual exorcisms or deliverance ministries. In clinical practice, it is important to understand internal conflicts or traumatic experiences which feed delusions of possession.

## Data Availability Statement

The original contributions generated for the study are included in the article/supplementary material, further inquiries can be directed to the corresponding author.

## Ethics Statement

The studies involving human participants were reviewed and approved by Ethical Review Board at the SWPS University of Social Sciences and Humanities in Poland. The patients/participants provided their written informed consent to participate in this study.

## Author Contributions

IP was responsible for project design and administration, collected and analyzed qualitative data, and prepared the manuscript. UK transcribed and analyzed interviews and participated in manuscript preparation. RT performed psychiatric consultations, helped in qualitative data analysis, and manuscript preparation. All authors contributed to the article and approved the submitted version.

## Conflict of Interest

The authors declare that the research was conducted in the absence of any commercial or financial relationships that could be construed as a potential conflict of interest.
